# Effects of Glide Path on the Centering Ability and Preparation Time of Two Reciprocating Instruments

**DOI:** 10.7508/iej.2016.01.007

**Published:** 2015-12-24

**Authors:** Marcelo Santos Coelho, Carlos Eduardo Fontana, Augusto Shoji Kato, Alexandre Sigrist de Martin, Carlos Eduardo da Silveira Bueno

**Affiliations:** a *Department of Endodontics, Centro de Pesquisa Odontológica, Faculdade de Odontologia São Leopoldo Mandic, Campinas, SP, Brazil*

**Keywords:** Centering Ability, Glide Path, Reciproc, Root Canal Preparation, Single-File Instrumentation, WaveOne

## Abstract

**Introduction:**

The aim of this *in vitro *study was to evaluate the effects of establishing glide path on the centering ability and preparation time of two single-file reciprocating systems in mesial root canals of mandibular molars.

**Methods and Materials:**

Sixty extracted mandibular molars with curvatures of 25-39 degrees and separate foramina for the mesiobuccal and mesiolingual canals, were divided into four groups (*n*=15); WaveOne+glide path; WaveOne; Reciproc+glide path and Reciproc. Non-patent canals were excluded and only one canal in each tooth was instrumented. A manual glide path was established in first and third groups with #10, 15 and 20 hand K-files. Preparation was performed with reciprocating in-and-out motion, with a 3-4 mm amplitude and slight apical pressure. Initial and final radiographs were taken to analyze the amount of dentin removed in the instrumented canals. The radiographs were superimposed with an image editing software and examined to assess discrepancies at 3-, 6- and 9-mm distances from the apex. The Kruskal-Wallis test was used for statistical analysis. The level of significance was set at 0.05.

**Results:**

Preparation in groups without glide paths was swifter than the other groups (*P*=0.001). However, no difference was observed regarding centering ability.

**Conclusion:**

Establishing a glide path increased the total instrumentation time for preparing curved canals with WaveOne and Reciproc instruments. Glide path had no influence on the centering ability of these systems.

## Introduction

Root canal preparation procedure must preserve the canal’s original anatomy by respecting its initial curvature and creating a continuously tapering funnel [1]. The shaping of curved canals remains a major challenge for clinicians, and is one of the most important steps of endodontic therapy [2]. Procedural errors occurring during preparation may lead to remaining debris and poor root canal cleaning/filling, and may also jeopardize the healthy structure of root dentin [3]. Before the development of engine-driven root canal preparation instruments, attaining a centralized manual preparation without ledges, zips or deviations was made easier after introduction of the balanced force concept by Roane *et al.* [4].

The increasing use of nickel-titanium (NiTi) engine-driven instruments has made shaping more predictable and less time-consuming. However, it still entails a long learning curve [5-9]. Preparation of curved canals is time-consuming and requires extensive training. File separation and other procedural mishaps are frequent, leading to a high risk of unsuccessful treatment outcomes [10-12].

A new concept of single-file root canal preparation has recently been introduced that does not necessitate primary hand instrumentation after canal negotiation with a #10 K-file and working length (WL) determination [13]. Instead of a continuous rotary motion, a reciprocating back and forth motion (similar to balanced force technique) is used to allow progression of the file within the canal, while minimizing the risk of instrument fracture due to cyclic fatigue [9]. This system requires a dedicated reciprocating motor to produce a counterclockwise motion that allows the instrument engagement with dentin, and a shorter clockwise motion to release the file from the dentin wall, thus allowing it to advance towards the apex [13-17]. In a way, this motion may be considered similar to balanced force, and allows the reciprocating instrument to reach the WL, in the majority of cases, without any prior preparation [13, 14]. The reciprocating motion leads to more centralized canal preparation in comparison with continuous rotation using the same instrument [18]. Reciproc (VDW, Munich, Germany) and WaveOne (Dentsply Maillefer, Baillagues, Switzerland) are single-instrument systems, and have recently been launched with similar motions and principles, but with different cross sections [19-21]. They use M-Wire alloy, which is 390% more resistant than the traditional Nitinol [22]. 

Reciproc has S-shaped cross-section, a non-cutting tip and sharp cutting edges that shapes the canal by means of a reciprocal back-and-forward motion with a speed of 300 rpm (150 degrees counterclockwise and then 30 degrees clockwise). This single file system is available at three different sizes and tapers; R25 (25/0.08), R40 (40/0.06) and R50 (50/0.05) [9]. WaveOne (Dentsply Maillefer, Ballaigues, Switzerland) is another single-file system with a reverse taper, variable helical angle and a non-active edge. It is used with 170^°^ counterclockwise rotation (direction of cutting) and 50^°^ clockwise rotation at a speed of 300 rpm. WaveOne is also available in different tip sizes and tapers; 21/0.06, 25/0.08 (primary) and 40/0.08 [9]. These new single-file techniques have the potential of reducing canal preparation time [23], while performing the similar shaping accomplished by full rotary sequences [20, 24]. 

The glide path is defined as a smooth patent pathway from the canal orifice to its physiologic terminus, which must be discovered when present or prepared when absent [25]. Early canal enlargement, up to a #20 file, may significantly reduce the risk of canal modifications and fracture of rotary instruments, mainly in curved canals [26, 27]. The manual or rotary creation of a glide path is an effective way to preserve root canal anatomy [28]. A simpler preparation procedure would allow less experienced professionals to achieve the same preparation level as that achieved by experts [7, 29]. 

Some studies have evaluated the influence of glide path establishment on preparation of resin blocks; nonetheless the behavior of these systems with and without previous glide path in extracted teeth is still unclear. The aim of this *in vitro* study was to evaluate the influence of a manual glide path on the centering ability and preparation time of two different single-file systems in curved mesial root canals of mandibular molars.

## Materials and Methods

***Specimen preparation***

After approval by the ethics committee of Faculdade de Odontologia São Leopoldo Mandic, Campinas, SP, Brazil (Registration No.: 2012/0124), 96 first and second extracted mandibular molars were selected. The teeth were radiographed with a buccal-to-lingual projection and selected after close analysis of their root curvature, according to Schneider’s method [30]. Only the roots with curvatures ranging from 25 to 39 degrees and separate foramina for the mesiobuccal and mesiolingual canals were included. Sixty mesial roots with fully formed apices were selected and divided into four groups (*n*=15). The teeth were sectioned at the cementoenamel junction with a diamond disk (Brasseler USA, Savannah, GA), leaving a standardized root length of 12 mm. The specimens were then stored in a 0.1% thymol solution (Fórmula e Ação, São Paulo, Brazil) until usage. Apical patency was checked by inserting a #10 K-file (Dentsply Maillefer, Ballaigues, Switzerland) into the root canals until its tip was visible at the apical foramen with unarmed eye, and WL was set 1.0 mm short of this measurement. Non-patent canals were excluded and only one canal in each tooth (either the mesiobuccal or mesiolingual canal) was instrumented. When patency was confirmed in both canals, the canal to be instrumented was randomly selected. The groups were randomly (www.random.org) assigned to the four groups: WaveOne Primary+glide path; WaveOne Primary; Reciproc R25+glide path; Reciproc R25.

The selected canal was initially irrigated with 5 mL of 2.5% sodium hypochlorite (NaOCl; Biodinâmica, São Paulo, Brazil) using a 31-G needle (Ultradent, South Jordan, UT) and a 10 mL plastic syringe. The canal was then explored with a #10 K-file. The glide path was created in groups 1 and 3 using #10, 15 and 20 hand K-files (Dentsply Maillefer, Ballaigues, Switzerland). The instruments were used until they were loose inside the canal, and each change of instrument was followed by irrigation with 2 mL of 2.5% NaOCl. Patency was re-checked in all specimens. The manual instruments were used for a maximum of three specimens, and were immediately replaced if any sign of distortion was observed under a dental operating microscope.

In all the groups, canal preparation was performed with reciprocating motion using appropriate settings of a gear reduction handpiece (Sirona Dental Systems GmbH, Bensheim, Germany) powered by a torque-controlled motor (Silver; VDW GmbH, Munich, Germany). All instruments, were used with an in-and-out pecking motion, with an amplitude of 3-4 mm and light apical pressure. After 3 motions, the instruments were removed and cleaned with gauze, and patency was checked once more with #10 hand K-file. Irrigation with 2 mL of 2.5% NaOCl was performed every time the instrument was re-inserted in the root canal. Instrument motion was repeated until the pre-established WL was achieved. Each instrument was used for a maximum of three roots, and observed under a dental operating microscope after each preparation. Should any sign of distortion occurred, the instrument was immediately replaced. All procedure was done by the same operator.

***Image analysis***

Pre- and post-operation radiographs were taken with similar positions, to analyze the amount of dentin removed from the canal walls. A device was constructed to hold the roots in a standardized position. The device consisted of a rectangular box made of medium density fiberboard (MDF), measuring 4×3 cm, which was filled with acrylic resin (Orthoplast, Zeist, The Netherlands) that held the roots in place. Before placing the roots in the resin, they were isolated with petroleum to prevent them from becoming permanently attached to the resin. Small portions of Coltosol (Coltene-Whaledent, Allstetten, Switzerland) were placed near the vertices of each MDF box to provide radiopaque landmarks serving as reference points to guide the precise superimposition of the initial and final radiographic images. Addition silicon impression material (Adsil; Coltene, Rio de Janeiro, Brazil) was used to ensure that the box and the specimens were kept in similar position for both initial and final radiographs, which were taken at a distance of 5 cm with an apparatus (Dabi-Atlante, Rio de Janeiro, Brazil) having the following exposure settings: 50 kVp, 7 mA and 0.2 sec of exposure time. A digital radiography sensor (Gendex Dental Systems, Hatfield, USA) was used to obtain the radiographic images in JPEG format.

The multiply blending option of Photoshop CS5 software (Adobe Systems Inc., San Jose, CA) was used to superimpose the initial and final images. Image size was set at 100% and contrast settings were changed when necessary to better visualize the canal margins. Measurement discrepancies were assessed at the distances of 3, 6 and 9 mm from the apex. Pre-and post-instrumentation measurements were recorded to evaluate the canal transportation and centering ratio based on the method described by Gambill *et al.* [6]. The dentin thickness was measured both in mesial and distal sides of roots at the distances mentioned above. The deviation ratio was obtained dividing the difference between the initial and final mesial side by the initial and final distal side. Where the initial mesial side is represented by Y1, the final mesial side is the Y2; on the other hand X1 and X2 represent specifically the initial and final measurements on the distal side, respectively [(Y1-Y2)/(X1-X2)]. The result closest to 1 was the most centralized one as it indicates that the preparation removed the same amount of dentin from the both sides.

***Preparation time***

Time of preparation was recorded using a digital chronometer considering two different time spans. The overall time included initial 5 mL irrigation with 2.5% NaOCl solution, canal exploration with a #10 K-file, glide path creation (in groups with glide path), reciprocating instrumentation and final irrigation with 5 mL of 17% EDTA accompanied by agitation with a #10 K-file for 60 sec followed by 3 mL of 2.5% NaOCl. The reciprocating time was recorded from the beginning of preparation with Reciproc or WaveOne instruments until the WL was reached. The chronometer was kept running if further instrument action was considered necessary after three initial motions, including the time required for cleaning the instrument, further irrigation and patency confirmation. The Kruskal Wallis test (Dunn Method) was used for statistical analysis with the level of significant set at 0.05

## Results

No statistically significant differences were found with regards to the centering ability between the four groups or among the 3 different distances from the apex (3, 6 and 9 mm), within each group (Table 1). No instrument fracture or signs of deformation was detected.

Groups with glide path had significantly longer total preparation times (*P*<0.05) (Table 2). However, when only the reciprocating instrumentation time was recorded, groups with glide path showed significantly shorter times (Table 3).

**Table 1:** Mean (SD) of canal centering in different canal sections

**Distance from apex**	**WaveOne+glide path**	**WaveOne**	**Reciproc+glide path**	**Reciproc**	***P *** **value**
**3 mm**	1.56 (0.81)	1.73 (1.58)	1.36 (1.12)	1.99 (2.20)	0.8727
**6 mm**	1.52 (1.38)	2.16 (2.56)	1.68 (1.01)	2.30 (1.42)	0.2585
**9 mm**	1.60 (1.00)	1.32 (0.98)	1.46 (0.91)	1.44 (1.06)	0.8649
***P *** **value**	0.5583	0.5363	0.4437	0.2333	

**Table 2:** Mean (SD) of total time (sec) required for canal preparation in study groups (Different superscript letters show a significant difference

**WaveOne+glide path**	**WaveOne**	**Reciproc+glide path**	**Reciproc**
269.37 (49.11)^a^	225.31 (47.04)^b^	269.85 (20.40)^a^	204.60 (18.59)^b^

**Table 3:** Mean (SD) of reciprocation time (sec) required for canal preparation in study groups (various superscript letters show a significant difference

**WaveOne+glide path**	**WaveOne**	**Reciproc+glide path**	**Reciproc**
49.58 (20.00)^a^	99.15 (46.06)^b^	48.46 (11.61)^a^	76.69 (19.20)^b^

## Discussion

The findings of the present study showed no difference between WaveOne and Reciproc regarding the centrality of prepared canals with or without glide path establishment. The two systems also performed similarly when the total preparation time was evaluated not considering the time required for glide path creation. The creation of glide path increased the total preparation time, but decreased the time required for both reciprocating systems to reach the WL. 

Recent studies on instrument-centering ability have been performed in artificial canals of acrylic resin blocks [7, 16, 17, 20, 21, 29, 31]. This material provides improved standardization of specimens, but also carries the disadvantage of not providing natural dentin hardness, structure and anatomy, preventing reliable extrapolation of the results to natural teeth [21]. In spite of the difficulty involved in standardization of the specimens, extracted teeth were used in the present study, as reported by other authors in the related literature [6, 14, 19, 23].

Recent studies have focused on the two-dimensional (2D) evaluation of preparation; however, in spite of providing a reproducible model this methodology has the limitation of not showing a three-dimensional (3D) evaluation [7, 21, 28, 29, 31]. The formula used in the present study is based on the formulation suggested by Gambill *et al.* [6] that used computed tomography for image acquisition but assessed a 2D cross-section of specimens. Using a mathematic formula, the authors of the present study could quantify the centering ability of the preparations which avoided the use of subjective assessment by different evaluators.

The procedure of making measurements at different distances from the canal apex has been adopted by other authors [21, 32]. This method was used to compare the influence of glide path establishment on Reciproc and WaveOne in simulated canals by Lim *et al.* [21].

Glide path preparation is well established as an important step before rotary instrumentation, which prevents instrument wear and its separation rate [26, 27, 33]. However, the role of a glide path for single-file reciprocating systems has yet to be fully understood. A recent study has shown better preparations for WaveOne when a glide path was created [17]. Another study found WaveOne Primary instrument to be superior to the ProTaper sequence up to file F2; in both systems, a glide path was also created [16]. On the other hand, another study has shown that both reciprocating systems without a glide path maintain the original canal curvature better than the ProTaper and Profile rotary systems [20]. In the present study, both systems had the same performance, and the creation of primary glide path had no influence on centering ability. Lim *et al.* [21] compared both systems with and without glide path using acrylic resin blocks. The results showed that, at the distances of 1 and 2 mm from the apex, glide path creation was significantly associated with better canal centrality when WaveOne was used. As a result, it has been recommended that WaveOne should be used with a glide path created with a file size larger than #15.

On the other hand, Yoo and Cho [20] have shown that both reciprocating systems without a glide path maintain the original canal curvature better than the ProTaper and Profile files. Buklein *et al.* [31] reported that glide path creation had no effect on the centering ability of WaveOne and Reciproc. Lim *et al.* [21] also showed that at 3-, 5- and 7-mm distances from the apex, establishment of glide path does not improve the centering ability of both systems. The present study is in accordance with these results.

Because canal patency was among inclusion criteria in our study, it was possible to reach the WL in all specimens. De-Deus *et al.* [14] evaluated the Reciproc R25 instrument in mandibular molars, and reached full WL in 90.66% of curved canals and 100% of those canals in which canal patency was checked with a #10 K-file.

Reciprocating angle and centering ability was the subject of a recent study [32]. A more centered preparation was detected with a smaller angle of reciprocation, but a longer preparation time was required. The manufacturers of both WaveOne and Reciproc fail to mention the exact range of reciprocation used in their motors [34]. Although no difference in centering ratio was detected in the present study, relevant discrepancies found in other studies have found that the reciprocating range is a factor influencing centering results. Hence, our opinion is that further investigation is warranted to establish the influence of reciprocating angles on centering ability and canal shaping. 

Preparation of a glide path decreased the time required to reach the WL, from 99.15 to 49.58 sec and from 76.69 to 48.46 sec, for WaveOne and Reciproc, respectively. Bürklein *et al.* [19] showed that Reciproc R25 was faster than WaveOne Primary. On the other hand, Park *et al.* [35] reported that preparation with WaveOne was faster than Reciproc. In the present study, Reciproc R25 without a glide path was faster than WaveOne, although the difference was not statistically significant. Both systems performed similarly when a glide path was present. Single-instrument root canal preparation is less time-consuming and more comfortable for both the patient and the clinician [13]. 

The present study found that a glide path had no influence on the centering ability of both WaveOne and Reciproc single-file systems. The time required by these systems to prepare curved canals decreased, but total preparation time increased; nevertheless, further investigation is warranted to ascertain the full extent of the role played by the preparation of a glide path on the shaping of curved root canals and on treatment outcomes when using these new systems. The null hypothesis that the glide path has no influence on centering ability is confirmed, but its influence over preparation time is rejected.

## Conclusion

Within the limitations of this study, a manual glide path increased the total time involved in preparation of curved canals with WaveOne and Reciproc instruments. A glide path had no influence on the centering ability of these systems.
